# Anti-Aβ Drug Screening Platform Using Human iPS Cell-Derived Neurons for the Treatment of Alzheimer's Disease

**DOI:** 10.1371/journal.pone.0025788

**Published:** 2011-09-30

**Authors:** Naoki Yahata, Masashi Asai, Shiho Kitaoka, Kazutoshi Takahashi, Isao Asaka, Hiroyuki Hioki, Takeshi Kaneko, Kei Maruyama, Takaomi C. Saido, Tatsutoshi Nakahata, Takashi Asada, Shinya Yamanaka, Nobuhisa Iwata, Haruhisa Inoue

**Affiliations:** 1 Center for iPS Cell Research and Application, Kyoto University, Kyoto, Japan; 2 Core Research for Evolutional Science and Technology, Japan Science and Technology Agency, Saitama, Japan; 3 Department of Pharmacology, Faculty of Medicine, Saitama Medical University, Saitama, Japan; 4 Laboratory for Proteolytic Neuroscience, RIKEN Brain Science Institute, Saitama, Japan; 5 Department of Morphological Brain Science, Graduate School of Medicine, Kyoto University, Kyoto, Japan; 6 Department of Neuropsychiatry, Institute of Clinical Medicine, University of Tsukuba, Tsukuba, Japan; 7 Yamanaka iPS Cell Special Project, Japan Science and Technology Agency, Saitama, Japan; 8 Graduate School of Biomedical Sciences, Nagasaki University, Nagasaki, Japan; Tokyo Medical and Dental University, Japan

## Abstract

**Background:**

Alzheimer's disease (AD) is a neurodegenerative disorder that causes progressive memory and cognitive decline during middle to late adult life. The AD brain is characterized by deposition of amyloid β peptide (Aβ), which is produced from amyloid precursor protein by β- and γ-secretase (presenilin complex)-mediated sequential cleavage. Induced pluripotent stem (iPS) cells potentially provide an opportunity to generate a human cell-based model of AD that would be crucial for drug discovery as well as for investigating mechanisms of the disease.

**Methodology/Principal Findings:**

We differentiated human iPS (hiPS) cells into neuronal cells expressing the forebrain marker, Foxg1, and the neocortical markers, Cux1, Satb2, Ctip2, and Tbr1. The iPS cell-derived neuronal cells also expressed amyloid precursor protein, β-secretase, and γ-secretase components, and were capable of secreting Aβ into the conditioned media. Aβ production was inhibited by β-secretase inhibitor, γ-secretase inhibitor (GSI), and an NSAID; however, there were different susceptibilities to all three drugs between early and late differentiation stages. At the early differentiation stage, GSI treatment caused a fast increase at lower dose (Aβ surge) and drastic decline of Aβ production.

**Conclusions/Significance:**

These results indicate that the hiPS cell-derived neuronal cells express functional β- and γ-secretases involved in Aβ production; however, anti-Aβ drug screening using these hiPS cell-derived neuronal cells requires sufficient neuronal differentiation.

## Introduction

Alzheimer's disease (AD) is the most common cause of dementia in the elderly. It is characterized clinically by progressive declines in memory, executive function, and cognition. It is also characterized by pathological features, including the deposition of amyloid plaques and neurofibrillary tangles as well as neuronal and synaptic loss in particular areas of the brain [1]. Accumulation of amyloid β peptide (Aβ) is hypothesized to initiate the pathogenic cascade that eventually leads to AD. The amyloid hypothesis is based on an imbalance between the production and clearance of Aβ [2]. Aβ is produced by β- and γ-secretase-mediated sequential proteolysis of amyloid precursor protein (APP) and plays a central role in AD pathogenesis. Because β- and γ-secretases are directly involved in Aβ production, they are straightforward and attractive therapeutic targets for AD. A number of compounds that inhibit or modulate these secretase activities and Aβ levels *in vitro* and *in vivo* have to date been developed [3], [4].

Development of a human, cell-based *in vitro* assay system is a basic requisite for drug discovery and for investigating mechanisms of the disease. Induced pluripotent stem (iPS) cells reprogrammed from somatic cells [5], [6] provide an opportunity to easily generate and use patient-specific differentiated cells. Because previous AD assay systems using human cancer cell lines or primary rodent cell cultures did not perfectly present the human intracellular environment or components, human iPS (hiPS) cell-derived neuronal cells may enable the development of more efficient drugs, such as γ-secretase modulators, and the better elucidation of AD mechanisms. In this study, we successfully generated forebrain neurons from hiPS cells, and showed that Aβ production in neuronal cells was detectable and inhibited by some typical secretase inhibitors and modulators. Thus, we provide a new platform for AD drug development, which might be applied to AD patient-specific iPS cell research.

## Results

### Differentiation of forebrain neurons from hiPS cells

Recently, forebrain neurons were successfully differentiated from mouse embryonic stem (ES) cells [7], [8], [9] and human ES and/or iPS cells [9], [10], [11]. The methods used for differentiation into spinal motor neurons and midbrain dopaminergic neurons required the morphogens retinoic acid (RA)/sonic hedgehog (SHH) and fibroblast growth factor 8 (FGF8)/SHH, respectively [11], [12]. On the other hand, non-morphogens [10], [11] or Lefty A and Dickkopf homolog 1 (Dkk1) [7], [9] have been used for the induction of hiPS cells into forebrain neurons. Because amyloid plaques are observed in the cerebral cortex from the early stage of AD development [13], stem cells should be differentiated to at least forebrain neurons for *in vitro* assays in AD research.

**Figure 1:**
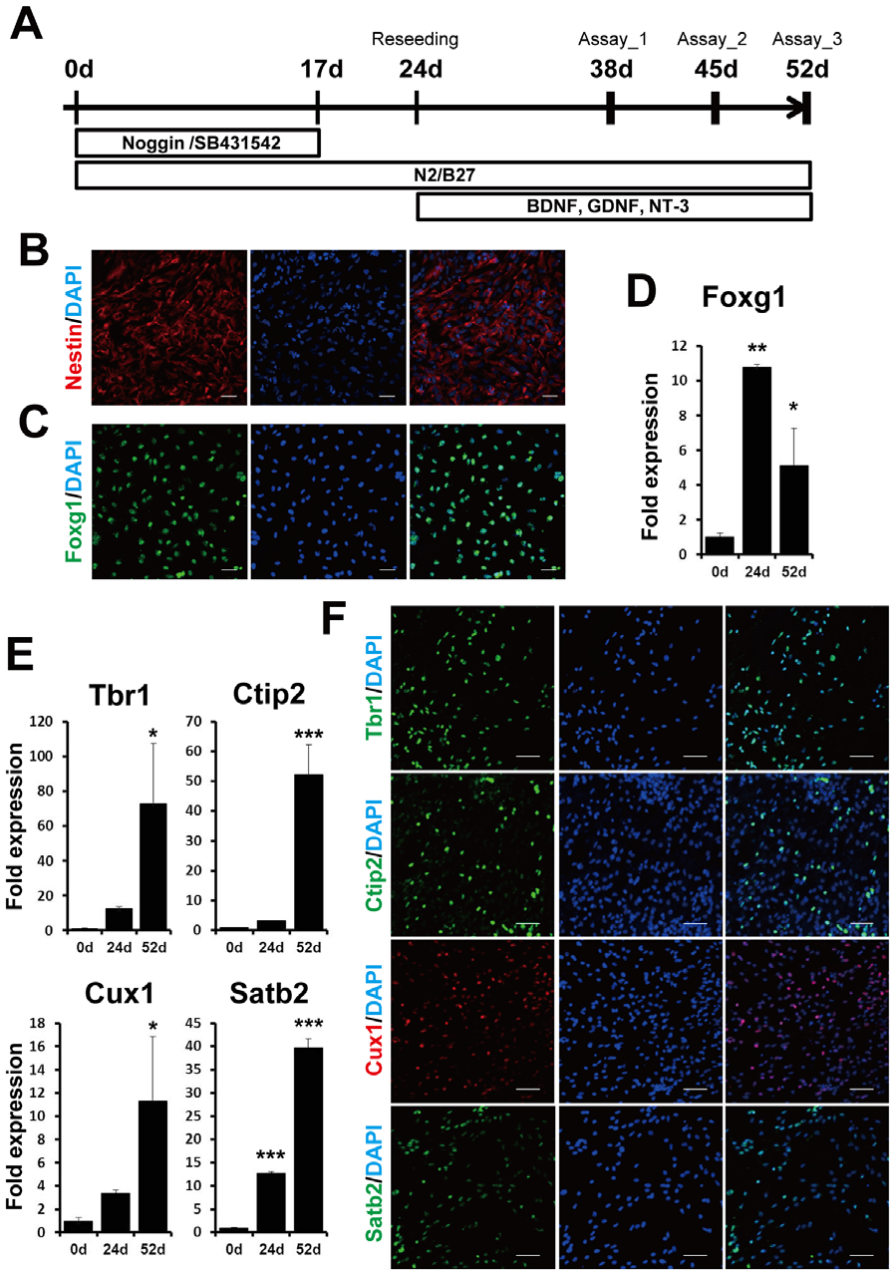
Differentiation of forebrain neurons from hiPS cells. (A) Experimental scheme of neural differentiation from hiPS cells, 253G4. Nestin-positive neuroepithelial cells (B) and Foxg1-positive cells (C) were observed at days 17 and 24, respectively. Scale bar, 50 µm. Expression levels of Foxg1 (D) and the neocortical markers Tbr1, Ctip2, Cux1, and Satb2 (E) at days 0, 24, and 52. Expression levels were measured by qPCR and normalized by that of GAPDH. “Fold expression” is shown as a ratio of day 24/day 0 or day52/day 0. Each column represents the mean ± SD of 3 assays. ^*^ *p*<0.05, ^**^ *p*<0.01, ^***^ *p*<0.001, significantly different from day 0 by Dunnett's test. (F) ICC staining of Tbr1-, Ctip2-, Cux1- and Satb2-positive cells at day 52. Scale bar, 50 µm.

We differentiated forebrain neurons from hiPS 253G4 cells, which were generated from human dermal fibroblasts using three reprogramming factors (Oct3/4, Sox2, and Klf4) [14], as described previously (Figure 1A) [12], [15]. When neural stem cells were induced with Noggin and SB431542 for 17 days, we obtained cells that were positive for the neuroectodermal marker, Nestin (Figure 1B), as previously reported using human and monkey ES cells [15]. After culturing the cells with morphogen-free medium for days 17–24, Forkhead box G1 (Foxg1) expression was induced and Foxg1-positive cells were observed (Figure 1C, D) [11], [15]. We also examined whether treatment with cyclopamine, an SHH inhibitor, increased the number of neurons presenting a glutamatergic phenotype as observed in mouse ES cells [8]. The expression level of vesicular glutamate transporter 1 (vGlut1), a glutamatergic marker, was not significantly increased by the addition of cyclopamine (final concentration 1 µM) from days 17 to 24 (data not shown). Therefore, we did not add cyclopamine in this period in subsequent experiments. At day 24, dissociated cells were reseeded on 24-well plates to further characterize the cells.

Next, we evaluated the hiPS cell-derived neuronal cells using four cortical layer-specific markers, T-brain-1 (Tbr1) and chicken ovalbumin upstream promoter transcription factor (COUP-TF)-interacting protein 2 (Ctip2) [9], [10], [11], and cut-like homeobox 1 (Cux1) and special AT-rich sequence-binding protein 2 (Satb2) [16]. Quantitative polymerase chain reaction (qPCR) revealed that expression levels of these markers were increased in a differentiation day-dependent manner (Figure 1E). At day 52, all four of these markers were visualized by immunocytochemistry (ICC) (Figure 1F). The percentages of marker-positive cells relative to the total number of cells were 62.2±2.9% for Tbr1, 11.9±3.0% for Ctip2, 82.6±5.0% for Cux1, and46.0±7.1% for Satb2. The population of each marker-positive cell was similar to that of data reported previously in human fetal brain around gestational week-20 [16]. In this experimental schedule, most cells expressed one or a few neocortical markers at day 52.

### Characterization of hiPS cell-derived neuronal cells

**Figure 2:**
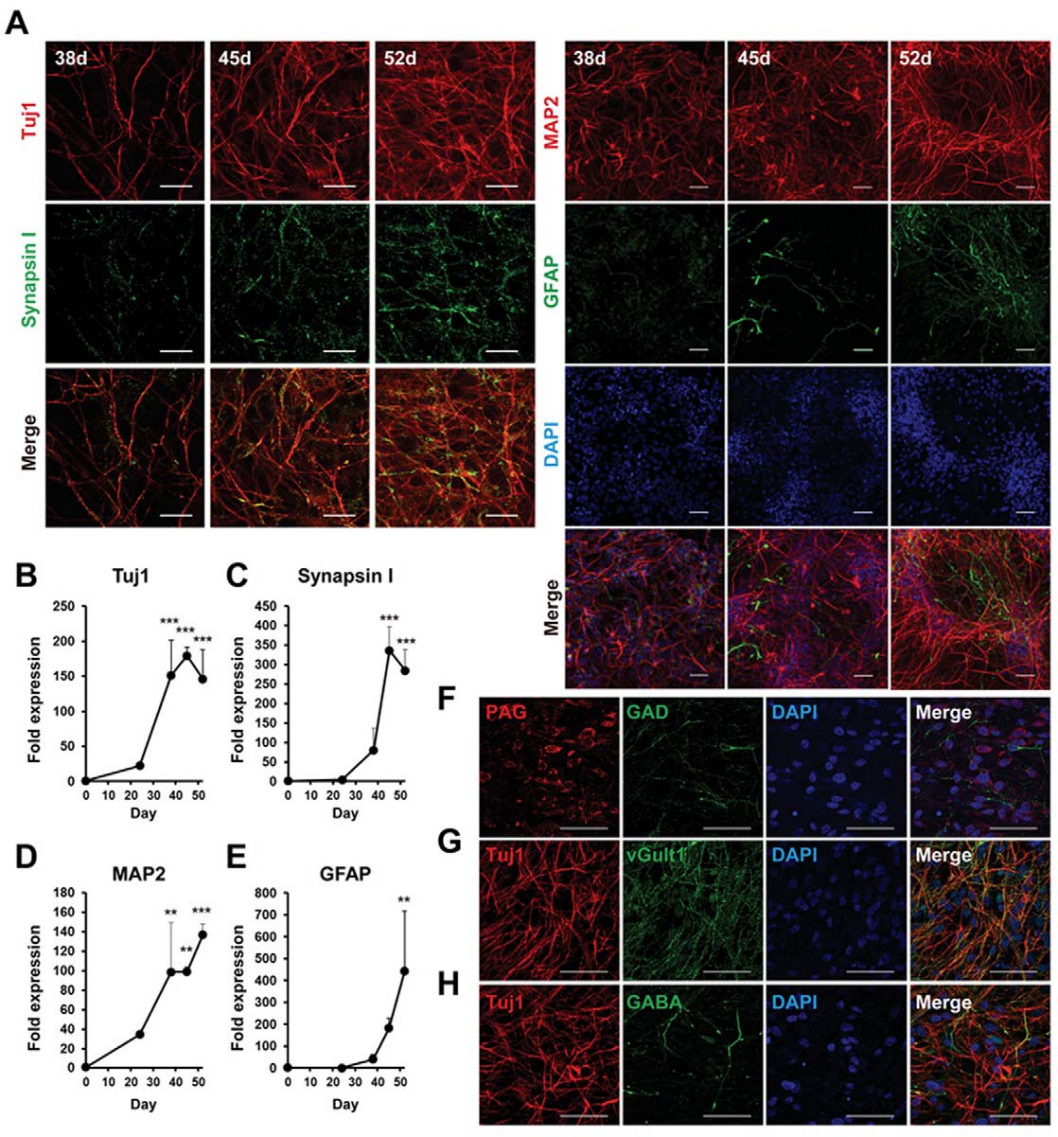
Characterization of neuronal and glial cells differentiated from hiPS cells. (A) Time-dependent morphological changes of cells reseeded in a 24-well plate. Neuronal and glial cells were stained by anti-Tuj1 (left; red), anti-synapsin I (left; green), anti-MAP2 (right; red), and anti-GFAP (right; green) antibodies and DAPI (right; blue) at 38, 45, and 52 days. Scale bar, left; 20 µm, right; 50 µm. Expression levels of Tuj1 (B), synapsin I (C), MAP2 (D), and GFAP (E) at days 0, 24, 38, 45, and 52 were measured by qPCR and normalized by that of GAPDH. “Fold expression” is the ratio of expression at each day compared to day 0. Each point represents mean ± SD of 3 assays. ^*^ *p*<0.05, ^**^ *p*<0.01, ^***^ *p*<0.001, significantly different from day 0 by Dunnett's test. (F–H) Neurotransmitter phenotypes at day 52. PAG (red)- and GAD (green)-positive (F), vGlut1 (green)- and Tuj1 (red)-positive (G), and GABA (green)- and Tuj1 (red)-positive cells (H). Blue, DAPI. Scale bar, 50 µm.

Cells that were reseeded at day 24, were sparsely adhered to the culture plate and had proliferated and extended neurites in a time course-dependent manner as observed by the neuronal marker, class-III β-tubulin (Tuj1), and microtubule-associated protein 2 (MAP2) (Figure 2A). Tuj1 expression was almost saturated at day 45 (Figure 2B), but MAP2 and synapsin I expression were still increasing (Figure 2C, D). Synaptic development continued until day 52, and many synapsin I-positive puncta were detected by ICC at day 52 (Figure 2A). Expression of the glial marker, glial fibrillary acidic protein (GFAP), was highest at day 52 in this schedule (Figure 2E). This sequential expression pattern is similar to that reported recently in human pluripotent stem cell-derived neurons; the synapsin I-positive neuronal and GFAP-positive glial cultures at day 52 corresponded to the stage at which spontaneous neuronal activity was observed [17].

We then examined the neurotransmitter phenotypes of these differentiated neurons by evaluating the synthesizing enzymes for two typical cortical neurotransmitters, glutamate and γ-aminobutyric acid (GABA). Expression of the glutamatergic neuronal marker, phosphate-activated glutaminase (PAG) [18], and the GABAergic neuronal marker, glutamate decarboxylase (GAD), were observed by ICC at day 52 (Figure 2F). PAG- and GAD-positive neurons comprised 60±20% and 5±4% of total cells, respectively. Most of the Tuj1-positive neurons were also co-localized with the punctate signals of vGlut1 (Figure 2G). GABA-positive neurons comprised a similar population to the GAD-positive ones (Figure 2F, H). On the other hand, cholineacetyltransferase (ChAT) or vesicular acetylcholine transporter (VAChT)-positive cholinergic neurons were little observed at day 52, although their mRNA level increased with differentiation time (Figure S1). These data showed that a majority of differentiated neuronal cells possessed a glutamatergic phenotype in the present condition.

### Differentiated neuronal cells express some components related to Aβ production

**Figure 3:**
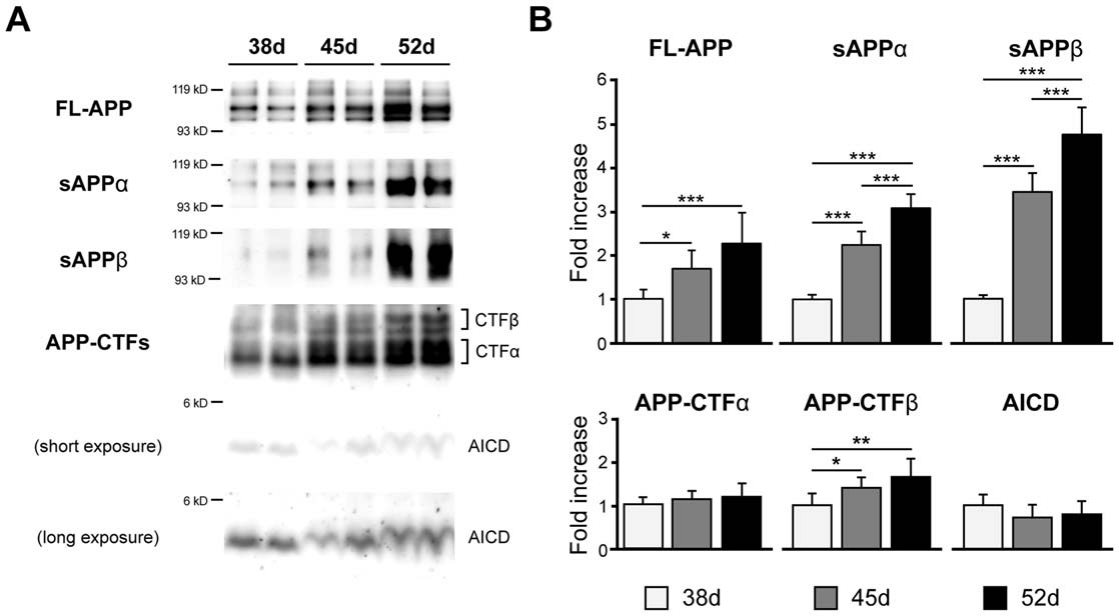
APP was expressed in hiPS cell-derived neuronal cells. HiPS cell-derived neuronal cells express full-length APP, sAPPα, sAPPβ, APP-CTFα, APP-CTFβ and AICD at 38, 45, and 52 days. (A) Representative western blots of APP and its fragments. (B) Each column represents mean ± SD of 8 samples measured by quantitative western blot analysis and normalized by that of β-actin. “Fold expression” represents the ratio of expression on the given day compared to day 38. ^*^ *p*<0.05, ^**^ *p*<0.01, ^***^ *p*<0.001, Tukey's test.

To evaluate their usefulness as an AD model, we measured the levels of Aβ secreted from the differentiated neuronal cells at days 38, 45, and 52. In the non-amyloidogenic pathway, α-secretase cleaves full-length APP (FL-APP) within the Aβ domain to the large soluble APP fragment (sAPPα) and APP-C terminal fragment α (CTFα) (Figure 3) [19]. In the amyloidogenic pathway, β-secretase, β-site APP cleaving enzyme 1 (BACE1), cleaves APP on the N-terminal side of the Aβ domain to soluble sAPPβ and APP-CTFβ (Figure 3). FL-APP and its cleavage products were increased in a time-course-dependent manner (Figure 3).

APP has three alternatively spliced isoforms: APP695, APP751, and APP770. APP695 is most abundantly expressed in neurons, whereas APP751 and APP770 show more ubiquitous expression patterns [20]. In cell lysates, we detected three separate APP variants on western blots. The estimated percentages of the neuron-dominant variant APP695 were 64.5±1.0%, 68.6±2.2%, and 69.6±2.1% at days 38, 45, and 52, respectively (Figures 3A and S2). The neuronal population at day 52 was approximately consistent with the sum of the percentages of the glutamatergic and GABAergic neurons mentioned above.

**Figure 4:**
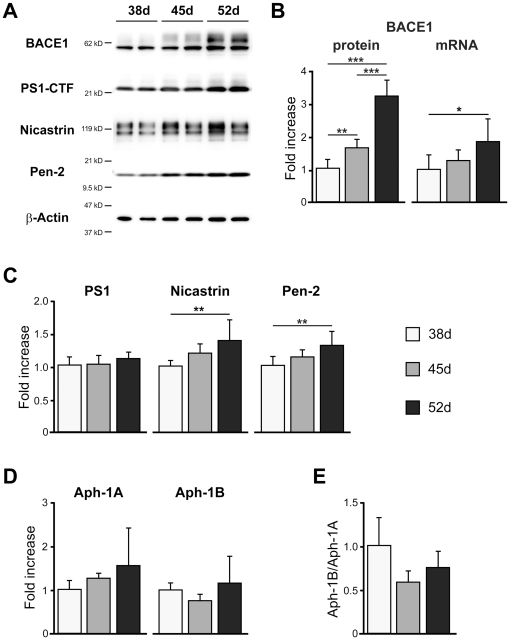
β-Secretase and γ-secretase components were expressed in hiPS cell-derived neuronal cells. The hiPS cell-derived neuronal cells express BACE1 protein and mRNA (B), γ-secretase components; presenilin 1(PS1), nicastrin, Pen-2 (C), and Aph-1A, and Aph-1B (D) at days 38, 45, and 52. Expression levels were quantified by western blot analysis (n = 8) (B, C) or qPCR (n = 3) (D) and normalized by that of β-actin. “Fold expression” represents the ratio of expression on the given day compared to day 38. (E) The ratio Aph-1B/Aph-1A. Data represent mean ± SD. (A) Representative western blots of BACE1 and γ-secretase components at 38, 45, and 52 days. ^*^ *p*<0.05, ^**^ *p*<0.01, ^***^ *p*<0.001, Tukey's test.

The aspartyl protease BACE1, the major β-secretase involved in cleaving APP, is a significant molecule for AD pathology because BACE1 protein levels and activity are increased in the brains of patients with the sporadic form of AD [21]. In our differentiated neurons, BACE1 protein levels were increased in a time course-dependent manner (Figure 4A, B), and we speculated that the upregulation of BACE1 protein levels may be due to a posttranscriptional mechanism [22]. BACE1 mRNA levels were slightly elevated with time (Figure 4B). These data may indicate that increased BACE1 protein levels were mainly induced by translational activation along with neuronal differentiation.

APP-CTFβ is cleaved to Aβ and APP intercellular domain (AICD) by γ-secretase (Figure 3). The γ-secretase complex consists of four core members, presenilin (PS; either PS1 or PS2), nicastrin, Pen-2, and Aph-1 [23]. PS1, nicastrin, and Pen-2 were detected by western blotting, but their expression levels did not change markedly over time (Figure 4A, C). Aph-1 has two isoforms in human, Aph-1A and Aph-1B, which are considered to have different effects on the production of Aβ species related to AD [24]. Their expression levels measured by qPCR were relatively constant (Figure 4D). The Aph-1B/Aph-1A ratios also did not show significant differences among the time points analyzed here (Figure 4E).

**Figure 5:**
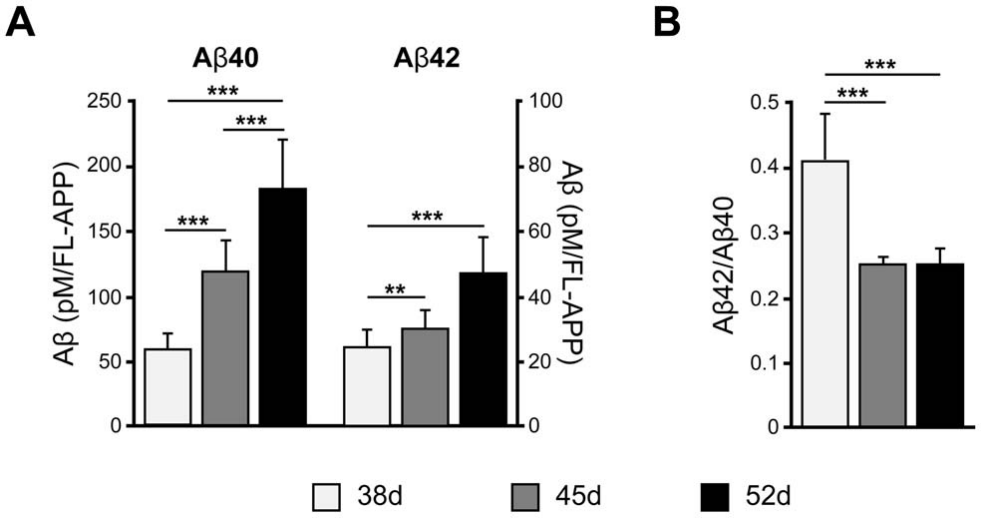
Aβ was produced in hiPS cell-derived neuronal cells. (A) Aβ40 or Aβ42 secreted into the conditioned media and FL-APP were measured by sandwich ELISA and western blot analysis, respectively. Expression level of Aβ was normalized by that of FL-APP. (B) Aβ42/Aβ40 ratios. Data represent the mean ± SD of 8 assays. ^*, #^ *p*<0.05, ^**, ##^ *p*<0.01, ^***, ###^ *p*<0.001, Tukey's test.

Aβ has several species, including Aβ40 and Aβ42, which have emerged as two of the most robust Aβ measurements in brain. Recent studies suggest that Aβ40 and Aβ42 may have different effects on Aβ aggregation or oligomerization [25], [26]. We measured Aβ40 and Aβ42 secreted into conditioned media for 2 days by sandwich ELISA. Both types of Aβ increased with time (Figure 5A). The level of Aβ40 was higher than that of Aβ42, compatible with previous reports [4], [27], [28], [29], [30]. Interestingly, the ratio of Aβ42/Aβ40 was highest at day 38, and there was no significant difference between days 45 and 52 (Figure 5B).

### Inhibition of Aβ40 and Aβ42 secretion

We examined whether the differentiated neurons contained funtional β- and γ-secretases and whether Aβ secretion could be controlled. We selected the most effective, commercially available β- and γ-secretase inhibitors, β-secretase inhibitor IV (BSI) [31] and γ-secretase inhibitor XXI/Compound E (GSI) [32], respectively. We also examined the effect of a non-steroidal anti-inflammatory drug (NSAID), sulindac sulfide [33], because some NSAIDs directly modulate γ-secretase activity to selectively lower Aβ42 levels [33], [34]. The cells were treated with each drug for 2 days, and Aβ was monitored in the collected media at day 38 or 52.

**Figure 6:**
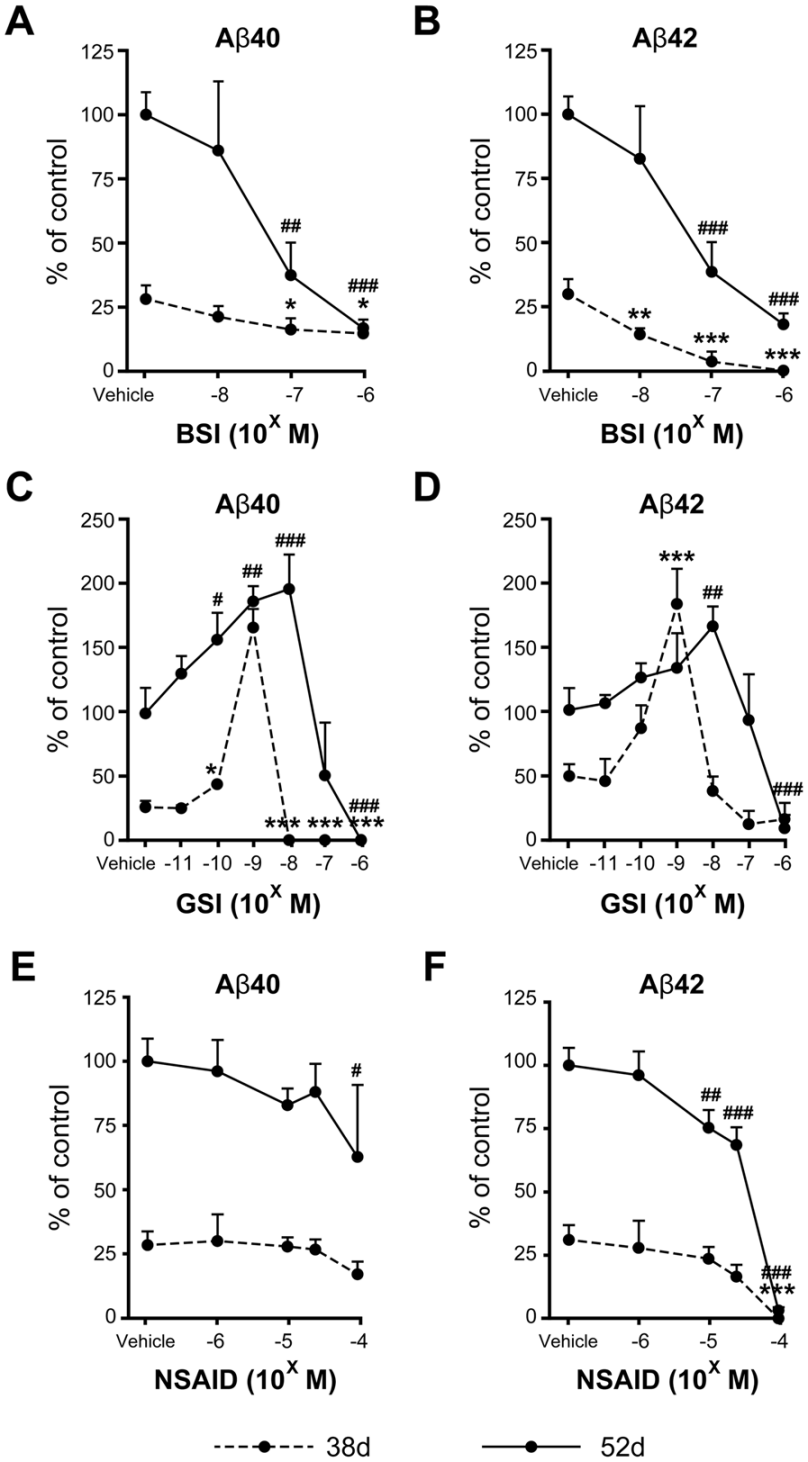
Aβ production was modulated by β- and γ-secretase inhibitors and an NSAID. β-Secretase inhibitor (BSI) (A, B), γ-secretase inhibitor (GSI) (C, D), and NSAID (E, F) were added into hiPS cell-derived neuronal cell cultures at day 36 (dotted line) and 50 (bold line), and two days later amounts of Aβ40 and Aβ42 secreted into the conditioned media were measured. The ratios Aβ40/FL-APP and Aβ42/FL-APP are expressed as percentages of the vehicle-treated group at day 52 and represent mean ± SD of 3 assays. A, B: There were significant main effects of day (F(1, 16) = 72.5 and 162.4, *p*<0.001 in Aβ40 and Aβ42, respectively) and dose (F(3, 16)  = 23.1 and 45.7, *p*<0.001 in Aβ40 and Aβ42, respectively), and significant interaction between day and dose (F(3, 16)  = 13.0 and 11.7, *p*<0.001 in Aβ40 and Aβ42, respectively) by 2-way ANOVA. C, D: There were significant main effects of day (F(1, 28)  = 240.5 and 59.1, *p*<0.001 in Aβ40 and Aβ42, respectively) and dose (F(6, 28)  = 70.8 and 37.8, *p*<0.001 in Aβ40 and Aβ42, respectively), and significant interaction between day and dose (F(6, 28)  = 23.5 and 15.1, *p*<0.001 in Aβ40 and Aβ42, respectively) by 2-way ANOVA. E, F: There were significant main effects of day (F(1, 20)  = 196.9 and 418.0, *p*<0.001 in Aβ40 and Aβ42, respectively) and dose (F(4, 20)  = 4.16, *p* = 0.013 and F(4, 20) = 91.9, *p*<0.001 in Aβ40 and Aβ42, respectively), and significant interaction between day and dose (F(4, 20) = 25.4, *p*<0.001 in Aβ42) by 2-way ANOVA. ^*, #^ *p*<0.05, ^**, ##^ *p*<0.01, ^***, ###^ *p*<0.001, significantly different from respective vehicle-treated groups by Dunnett's test.

There were different susceptibilities to all three drugs between days 38 and 52 (Figure 6) as revealed by two-way analysis of variance (ANOVA) [significant interaction between day and dose (BSI, *p*<0.001 in Aβ40 and Aβ42, respectively; GSI, *p*<0.001 in Aβ40 and Aβ42, respectively; NSAID, *p*<0.001 in Aβ42)]. Following BSI and NSAID treatment, secretion of Aβ40 and Aβ42 was decreased in a dose-dependent manner (Figure 6A, B, E, and F). NSAID especially showed more efficient inhibition of Aβ42 than that of Aβ40, consistent with a previous report [33]. Following GSI treatment (Figure 6C, D), secretion of both Aβ40 and Aβ42 was increased at lower doses (10^−11^–10^−8^ M), but was inhibited at higher doses (10^−7^–10^−6^ M) at day 52. This phenomenon, which is called a “gradual Aβ rise”, was observed following the addition of other GSIs in a cell line system [35]. On the other hand, secretion of both Aβ40 and Aβ42 at day 38 showed a fast increase at lower doses (10^−11^–10^−9^ M) (Aβ surge) and drastic decline at 10^−8^ M. We also examined the effects of these inhibitors on cell viability using the lactate dehydrogenase (LDH) assay. Two-day-treatments with the highest concentrations of BSI, GSI, or NSAID did not induce cell death (Table S1). We also traced these experiments using human ES (hES) cell (H9)-derived neuronal cells (Figure S4) because remaining expression of reprogramming factors, Oct3/4 and Klf4, were observed in hiPS cell (253G4)-derived neuronal cells (Figure S6). The Aβ production and its inhibition by these drugs in hES cell-derived neuronal cells were relatively similar to those in hiPS cell-derived ones (Figure S5). These data showed that BSI, GSI, and NSAID partially or fully blocked Aβ production in the hiPS cell-derived neuronal cells, indicating that these cells expressed functional β- and γ-secretases.

## Discussion

AD is the most common cause of dementia in the elderly, with progressive neuronal loss in the cerebral cortex and hippocampal formation. Although the underlying etiology of most AD remains unclear, Aβ is thought to play a pivotal role in its pathogenesis. Studies from animal and cellular models have shown that mutations in the APP, PS1, and PS2 genes affected the production of Aβ, which contributes to the formation of amyloid plaques [19]. In several strains of mouse models, Aβ levels in brain tissue, cerebrospinal fluid (CSF), and plasma have been associated with AD pathogenesis and cognitive impairment [27], [28], [36]. Human samples from clinical AD patients have also been used for pathological and biochemical analyses to understand the etiology of AD. Aβ levels in CSF and plasma have been examined to evaluate their risks for AD [29], [37], but brain tissues are only available postmortem for such analyses. On the other hand, immortalized human cell lines derived from kidney or brain, primary neurons derived from mice and rats, or cells artificially overexpressing APP or presenilin with or without familial AD mutations have been utilized for *in vitro* studies [4], [30]. There is no doubt that these cells are quite different from living neurons in the human body in terms of innate qualities. Although we have had no choice until recently, important advances in technology of iPS cells may now provide the opportunity to use intact human-derived neuronal cells [38].

We evaluated whether iPS cell-derived neuronal cells could be applied to an *in vitro* cell-based assay system for AD research. In particular, further investigations into the metabolic mechanisms of Aβ are requisite for drug development to treat the brains of patients afflicted with AD. In this respect, we provide a profile of the molecular components associated with Aβ production in hiPS cell-derived neuronal cells and propose to add an Aβ assay system using these cells to the panel of generalized Aβ-monitoring systems (Table 1). Human neuronal cells are considered to provide more accurate human neuronal conditions within which to evaluate drug efficacy or toxicity than other human cell lines (e.g., cancer lines). Furthermore, we would be able to investigate how hiPS cell-derived neuronal cells reflect AD-related physiological and pathological conditions based on Aβ production.

**Table 1:** Panel of Aβ monitoring systems.

Human sample	Aβ40	Aβ42	Ref.
Brain tissue [AD]	↑(AD/NC)	↑(AD/NC)	[39]
CSF [AD]	-→ (AD/NC)	↓(AD/NC)↓(AD/NC)	[37] [29]
Plasma [AD]	↑(AD/NC)	→ (AD/NC)	[29]
iPS cell-derived neuronal cells	Measurable	Measurable	This report

Mouse model	Aβ40	Aβ42	Ref.
Brain [PDAPP]Brain [APP23]Brain [Tg2576]	↑(Aging)↑(Tg/non-Tg)↑(Aging)↑(Tg/non-Tg)	↑(Aging)↑(Tg/non-Tg)↑(Aging)↑(Tg/non-Tg)	[36] [27] [28]
CSF [Tg2576]	↓(Aging)	↓(Aging)	[28]
Plasma [Tg2576]	↓(Aging)	↓(Aging)	[28]

Cell line	Aβ40	Aβ42	Ref.
[APP_NL_-H4][CHO-APP_NL_/SH-SY5Y-APP]	MeasurableMeasurable	MeasurableMeasurable	[30] [4]

AD, Alzheimer's disease; NC, normal control; Tg, transgenic mouse model.

In the present study, we characterized iPS cell-derived neuronal cells in terms of their expression of neuronal and glial markers by exposing them to Noggin and SB431542 during their differentiation (Figures 1 and 2). We observed increases in GFAP mRNA levels and in synapsin I-positive synaptic puncta at day 52. This was consistent with data showing that the existence of astrocytes promotes synaptic activity in human ES cell-derived neurons [40]. When differentiation occurred in the presence of non-morphogens, we obtained mainly glutamatergic neurons (Figure 2F, G), quite in line with previous reports of concerning hES and hiPS cells [10], [11]. Expression of the forebrain marker Foxg1 suggests a default forebrain identity of the 253G4 iPS cells used in this study (Figure 1C, D). We also observed the expression of the neocortex-specific transcriptional factors Tbr1, Ctip2, Cux1, and Satb2 (Figure 1E, F). These expression schemes appear to mimic human neocortical development *in vitro* [16], although further analyses are needed to assist in understanding human neuronal subtype-specific differentiation.

This is the first study to observe the expression of APP, β- and γ-secretase, and the production of Aβ in hiPS cell-derived neuronal cells. APP, sAPPβ, APP-CTFβ and BACE1 protein levels were increased (Figures 3 and 4), but protein levels of γ-secretase components were not significantly different during the period from day 38 to 52 (Figure 4C, D). Aβ production in hiPS cell 253G4-derived neuronal cells increased with differentiation course (Figure 5A), however that in another hiPS cell 201B7 [5]- and in hES H9-derived neuronal cells did not increase (Figures S5 and S7) although all cell lines showed development of synapse (Figure S4A) as Aβ releasing site [41], indicating that besides synaptogenesis, subtle changes in localization and assembly of APP [42], BACE1, γ-secretase components would be critical for Aβ production.

The Aβ42/Aβ40 ratio unexpectedly showed a significant decrease from day 38 to 45 (Figure 5B). Serneels *et al.* reported that the γ-secretase complex containing Aph-1B was active and involved in the generation of amyloidogenic Aβ42 [24]. Our data showed that the Aph-1B/Aph-1A ratio did not change significantly with cell differentiation (Figure 4E); therefore, the Aβ42/Aβ40 ratio may be influenced by other unknown factors interacting directly or indirectly with γ-secretase.

BSI, GSI, and the NSAID sulindac sulfide inhibited Aβ production in this human neuronal cell system (Figure 6). The inhibitory effect on Aβ production by GSI showed a characteristic difference between days 38 (Aβ surge) and 52 (gradual Aβ rise) (Figure 6C, D). Aβ surge at day 38 was also observed in another hiPS cell (201B7)-derived neuronal cells (Figure S7) as well as in hES cell line, H9-derived ones (Figure S5). At day 38, GSI might promote neuronal differentiation with synaptogenesis via blocking Notch signaling [43] rather than inhibition of Aβ production, leading to Aβ surge. Another possible explanation for Aβ surge is that change in conformation or components of the γ-secretase affects the sensitivity of γ-secretase to GSI (total Aβ, Aβ40, Aβ42, and Aβ42/Aβ40), although levels of mRNA and the ration for Aph-1A and Aph-1B do not change between days 38 and 52 (Figure 4D, E) Thus, for precise Aβ monitoring in human stem cell-derived neuronal cells, it is necessary to use neuronal cells with a sufficient substrate level and synaptogenesis, because Aβ is released presynaptically, as mentioned above.

Some NSAIDs are known to preferentially lower Aβ42 [33], [34]. Our data showed that sulindac sulfide was capable of inhibiting Aβ42 secretion at high concentrations (≥10^−5^ M) (Figure 6F), although a few NSAIDs do not show therapeutic effects for AD. Negative results might be due to low γ-secretase modulator potency [44]. To discover novel effective drugs for modulating β- or γ-secretase activity, the *in vitro* hiPS cell-derived neuronal cell assay system might be expected to yield such drugs.

Familial AD patient specific neuronal cells generated by direct conversion (induced neuron, iN) show higher Aβ42/Aβ40 ratio than those of unaffected individuals [45]. Based on this report, hiPS/hES cell-derived neurons expressing mutant PS1, PS2, or APP may show higher Aβ42/Aβ40 ratio. Comparing to our results, the levels of Aβs in this assay (Aβ40; ∼1.7 ng/ml at day 52) is higher than that using iN cells (Aβ40; ∼0.1 ng/ml), although iN cells become functional neurons more quickly. The optimization of neuronal cell condition for comparison of the Aβ42/Aβ40 ratio between multiple iPS cell-derived neuronal cells may be required.

In conclusion, our findings indicate that hiPS cell-derived neuronal cells express functional β- and γ-secretases related to the production of Aβ in the present experimental conditions. In addition, our data provide the proof in principle that hiPS cell-derived neuronal cells can be applied to drug screening and AD patient-specific iPS cell research.

## Materials and Methods

### Antibodies and reagents

Primary antibodies used were as follows: mouse anti-Nestin (1∶200, Millipore, Temecula, CA), mouse anti-Tuj1 (1∶2000, Covance, Princeton, NJ), rabbit anti-GFAP (1∶500, DAKO, Carpinteria, CA), rabbit anti-Synapsin I (1∶500, Millipore), mouse anti-Cux1 (1∶100, Abnova, Taipei, Taiwan), rabbit anti-Satb2 (1∶1000, Abcam, Cambridge, UK), rat anti-Ctip2 (1∶500, Abcam), rabbit anti-Tbr1 (1∶500, Abcam), rabbit anti-vGlut1 (1∶1000, Synaptic Systems, Göttingen, Germany), rabbit anti-Foxg1 (1∶100, Abcam), rabbit anti-GABA (1∶1000, Sigma-Aldrich, St. Louis, MO), rabbit anti-GAD65/67 (1∶200, Millipore), mouse anti-PAG [46] (1∶500), rabbit anti-APP (1∶15000, Sigma-Aldrich), mouse anti-APP (1 µg/ml, Millipore), rabbit-anti BACE (1∶2000, Merck, Darmstadt, Germany) mouse anti-PS1 loop C-terminus (1∶1000, Millipore), rabbit anti-nicastrin (1 µg/ml, Thermo Scientific, Rockford, IL), rabbit anti-Pen-2 (1∶1000, Invitrogen, San Diego, CA), mouse anti-MAP2 (1∶200, Millipore), goat anti-ChAT (1∶100, Millipore), guinea pig anti-VAChT (1∶500, Millipore), and mouse anti-β-actin (1∶15000, Sigma-Aldrich). We raised rabbit polyclonal antibodies against the carboxyl terminals of human sAPPα (hsAPPα) and sAPPβ using the KLH-conjugated synthetic peptides CRHDSGYEVHHQK and CKTEEISEVKM, respectively (Figure S3). All animal experiments were performed in compliance with the institutional guidelines at RIKEN Brain Science Institute, and were approved by the Animal Care and Use Committee (Permit number: H17-2B031). Each antibody was purified with a peptide-conjugated column [47]. Alexa Fluor 488 and Alexa Fluor 594-conjugated secondary antibodies (Invitrogen) were used for immunofluorescence.

The β-secretase inhibitor IV [31] and γ-secretase inhibitor XXI/Compound E [32] were purchased from Merck. Sulindac sulfide (NSAID) was purchased from Sigma-Aldrich.

### Immunocytochemistry

Cells were fixed with 4% paraformaldehyde in phosphate buffered saline (PBS) for 30 min, and incubated in PBS containing 0.2% Triton X-100 for 10 min (permeabilization). After blocking with 2% BSA in PBS, cells were incubated with primary antibody diluted with blocking buffer and then washed with PBS. Finally the cells were incubated with secondary antibodies and mounted using ProLong Gold antifade reagent with DAPI (Invitrogen). The immunoreactive cells were visualized using an LSM 700 Laser Scanning Microscope (Carl Zeiss, Jena, Germany) and a Biorevo BZ-9000 fluorescence microscope (Keyence, Osaka, Japan).

### Quantitative real-time RT-PCR

Total RNA was isolated from cells using TRIZOL reagent (Invitrogen). Contaminating DNA was removed using the TURBO DNA-free kit (Ambion, Austin, TX), and cDNA was synthesized using ReverTra Ace-α (Toyobo, Osaka, Japan), according to the manufacturers' protocols. Real-time PCR was performed using the StepOnePlus system (Applied Biosystems) and SYBR green reagent (TAKARA, Shiga, Japan). The primers used are listed in Table S2 in the supporting information.

### HiPS cell culture and differentiation into neuronal cells

HiPS cells, 253G4 [14] (passage 20–30) or hES cells, H9 were cultured on mitomycin C-treated mouse embryonic fibroblasts in primate ES medium (ReproCELL, Kanagawa, Japan) supplemented with bFGF (Wako Pure Chemicals, Osaka, Japan). To obtain cortical neurons derived from iPS cells, we partially modified a previous method [12], [15]. For neural induction, partially dissociated iPS cell colonies, 40–100 µm in diameter, were selected with Cell Strainer (BD Falcon, BD Bioscience, Bedford, MA) and plated on poly-L-lysine (Sigma-Aldrich)/Laminin (BD Biosciences) (PLL/LM)-coated dishes (P1) in N2B27 neuronal differentiation medium [DMEM/F12 (Invitrogen), Neurobasal (Invitrogen), N2 (Invitrogen), B27 minus vitamin A (Invitrogen), L-Gln (Invitrogen)], supplemented with 100 ng/ml human recombinant Noggin (R&D Systems, Minneapolis, MN) and 1 µM SB431542 (Sigma-Aldrich) for 17 days. At day 10, primary colonies were split into small clumps using 200 U/ml collagenase with CaCl_2_ and plated into PLL/Entactin-Collagen IV-Laminin (Millipore) (ECL)-coated dishes (P2). At day 17, P2 cells were dissociated using Accutase (Innovative Cell Technologies, San Diego, CA) and cultured on PLL/ECL-coated dishes (P3). Finally, at day 24, cells dissociated with Accutase were passed through a 40-µm cell strainer (BD Biosciences), counted, and cultured on PLL/LM/Fibronectin (Millipore)-coated 24-well plates at 2.5×10^4^ cells/well in N2B27 medium supplemented with 10 ng/ml BDNF, GDNF, and NT-3 (R&D Systems). Medium changes for cell culture were carried out once every two or three days until day 52.

### Aβ sandwich ELISA

At days 38, 45, and 52, two-day incubated conditioned media were collected from cultured neuronal cells and centrifuged at 4,000 *g* for 10 min. The resultant clear supernatants were subjected to sandwich ELISA (Wako) with a combination of monoclonal antibodies specific to the midportion of Aβ and specific to the C-terminal of Aβ40 or Aβ42, to determine the amounts of secreted Aβ, as described previously [20], [28], [30]. We also examined the inhibitory effect of each drug on Aβ production. All media were replaced with new media containing each drug and two-day conditioned media were analyzed as mentioned above.

### Western blot analysis

Western blot analysis was performed as previously described with minor modification. In addition to conditioned media, cell lysates were also collected, extensively washed with PBS, and lysed directly with 1× sample buffer (EzApply; ATTO, Tokyo, Japan). The media or cell lysates were separated by 5–20% gradient or 7.5% [FL-APP] or 10% [β-actin] sodium dodecyl sulfate-polyacrylamide gel electrophoresis (SDS-PAGE) and transferred to polyvinylidene difluoride membranes (Hybond-P; GE Healthcare, Buckinghamshire, UK). The blots were probed with an appropriate primary antibody, followed by HRP-conjugated anti-mouse or anti-rabbit IgG (GE Healthcare). The protein bands were visualized using an enhanced chemiluminescence (ECL) detection method (GE Healthcare), and band intensity was analyzed with a densitometer (LAS-4000; GE Healthcare), using the Science Laboratory 2001 Image Gauge software (Fujifilm, Tokyo, Japan). Immunoreactive protein content in each sample was calculated based on a standard curve constructed with each recombinant protein or one of the samples. Each set of experiments was repeated at least two times to confirm the results. The level of β-actin protein, measured by quantitative western blotting using β-actin antibody, was used as an extraction and loading control.

### LDH assay

Cell toxicity assays were performed using a cytotoxicity detection kit (LDH, Roche, Mannheim, Germany) according to the manufacturer's protocol.

### Statistical analysis

All data were expressed as mean ± SD. Comparisons of mean among more than three groups were done by one-way or two-way ANOVA, followed by *post*-*hoc* test (PRISM, GraphPad software). *P* values ≤0.05 indicated significant differences.

## Supporting Information

Figure S1 **Cholinergic neuronal marker-positive cells were observed in hiPS cell-derived neuronal cells.** Expression levels of ChAT (A) and VAChT (B) were quantified by qPCR (n = 3) and normalized by that of GAPDH. “Fold expression” represents the ratio of expression on the given day compared to day 38. ChAT- (C) and VAChT (D)-positive cells were observed a little at day 52.
(TIF)

Figure S2 **Percentages of the three isoforms of APP (APP770, APP751, and APP695) at 38, 45, and 52 days.** Each column represents mean ± SD of 8 assays. ^*^ *p*<0.05, ^**^ *p*<0.01, ^***^ *p*<0.001, Tukey's test.
(TIF)

Figure S3 **New hsAPPα and sAPPβ antibodies specifically detect human sAPPα and sAPPβ by western blots, respectively.** Human neuroglioma H4 cells overexpressing wild-type APP (APP_WT_-H4 cells) were treated with α-secretase activator (12-*O*-tetradecanoylphorbol 13-acetate (TPA)), α-secretase inhibitor (TNF-α protease inhibitor-2 (TAPI-2)), or β-secretase inhibitor (see Protocol S1). Brain lysates of APP-knockout mice (APP-KO) were used as negative control. Immunoblots of conditioned media and supernatants of brain lysates were probed by anti-hsAPPα or anti-sAPPβ antibody. sAPPα or sAPPβ derived from both exogenous APP695 and endogenous APP770/751 are detected by each antibody. The increase in sAPPα by α-secretase activator and the reduction in sAPPα by α-secretase inhibitor effectively reached 434% and 50% of control (DMSO), respectively (upper panel). The decrease in sAPPβ by β-secretase inhibitor effectively reached 11% of control (lower panel). Neither sAPPα nor sAPPβ in the APP-KO brain was detected by anti-hsAPPα or anti-sAPPβ antibody, respectively. An asterisk indicates a non-specific band.
(TIF)

Figure S4 **Immunocytochemical characterization of human ES cell (H9)-derived neuronal cells.** (A) Time-dependent morphological changes of cells reseeded in a 24-well plate. Neuronal and glial cells were stained by anti-Tuj1 (left; red), anti-synapsin I (left; green), anti-MAP2 (right; red), and anti-GFAP (right; green) antibodies and DAPI (right; blue) at 38, 45, and 52 days. Scale bar, left; 20 µm, right; 50 µm. (B) ICC staining of Tbr1-, Ctip2-, Cux1- and Satb2-positive cells at day 52. (C–E) Neurotransmitter phenotypes at day 52. PAG (red)- and GAD (green)-positive (C), Glut1 (green)- and Tuj1 (red)-positive (D), and GABA (green)- and Tuj1 (red)-positive cells (E). Blue, DAPI. Scale bar, 50 µm.
(TIF)

Figure S5 **Aβ production was modulated by several drugs in human ES cell-derived neuronal cells.** β-Secretase inhibitor (BSI) (A, B), γ-secretase inhibitor (GSI) (C, D), and NSAID (E, F) were added into hES cell-derived neuronal cell cultures at day 36 (dotted line) and 50 (bold line), and two days later amounts of Aβ40 and Aβ42 secreted into the conditioned media were measured. The ratios Aβ40/FL-APP and Aβ42/FL-APP are expressed as percentages of the vehicle-treated group at day 52 and represent mean ± SD of 3 assays. ^*, #^ *p*<0.05,^**, ##^ *p*<0.01, ^***, ###^ *p*<0.001, significantly different from respective vehicle-treated groups by Dunnett's test.
(TIF)

Figure S6 **Expression levels of reprogramming factors of iPS cells in neural differentiation.** Total and transgene (Tg) expression levels of Sox2, Oct3/4 and Klf4 were measured by qPCR. Bold and dotted lines represent total and transgene expressions, respectively. “Fold expression” represents the ratio of the expression level compared to the total expression level at day 0 (iPS cells).
(TIF)

Figure S7 **Aβ production was modulated by GSI in human iPS cell (201B7)-derived neuronal cells.** γ-Secretase inhibitor (GSI) was added into the hiPS cell line, 201B7-derived neuronal cell cultures at day 36 (dotted line) and 50 (bold line), and two days later amounts of Aβ40 (A) and Aβ42 (B) secreted into the conditioned media were measured. The ratios Aβ40/FL-APP and Aβ42/FL-APP are expressed as percentages of the vehicle-treated group at day 52 and represent mean ± SD of 3 assays.
(TIF)

Protocol S1 **Sampling method for checking antibody specificity.**
(PDF)

Table S1 **Effects of secretion inhibitors on cell viability measured by LDH assay at day 52.**
(DOCX)

Table S2 **qPCR primers.**
(DOCX)
