# Supplementary figures and images for: Anti-Aβ Drug Screening Platform Using Human iPS Cell-Derived Neurons for the Treatment of Alzheimer's Disease
Source: PLoS One. 2011 Sep 30;6(9):e25788. doi: 10.1371/journal.pone.0025788 (PMC3184175; doi:10.1371/journal.pone.0025788)

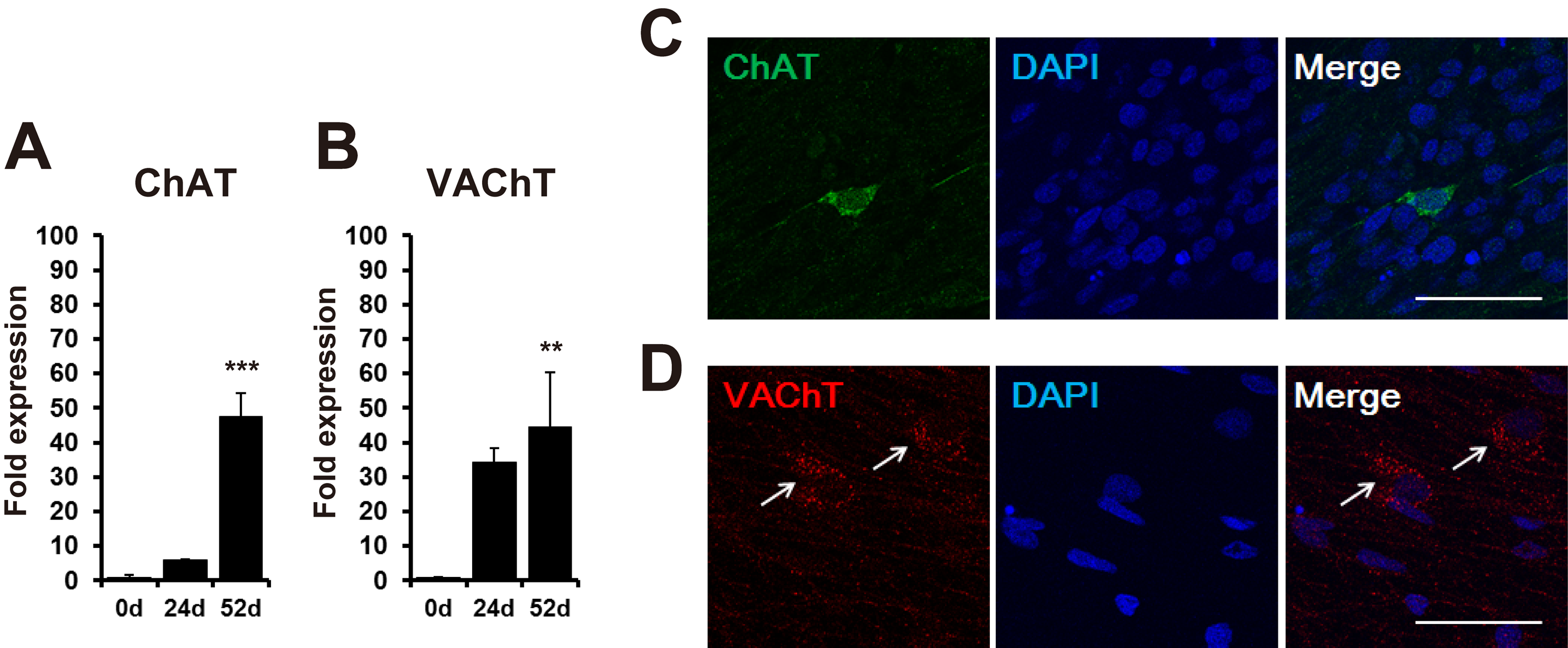

Supplement: Figure S1 — Cholinergic neuronal marker-positive cells were observed in hiPS cell-derived neuronal cells. Expression levels of ChAT (A) and VAChT (B) were quantified by qPCR (n = 3) and normalized by that of GAPDH. “Fold expression” represents the ratio of expression on the given day compared to day 38. ChAT- (C) and VAChT (D)-positive cells were observed a little at day 52. (TIF) [file pone.0025788.s001.tif]

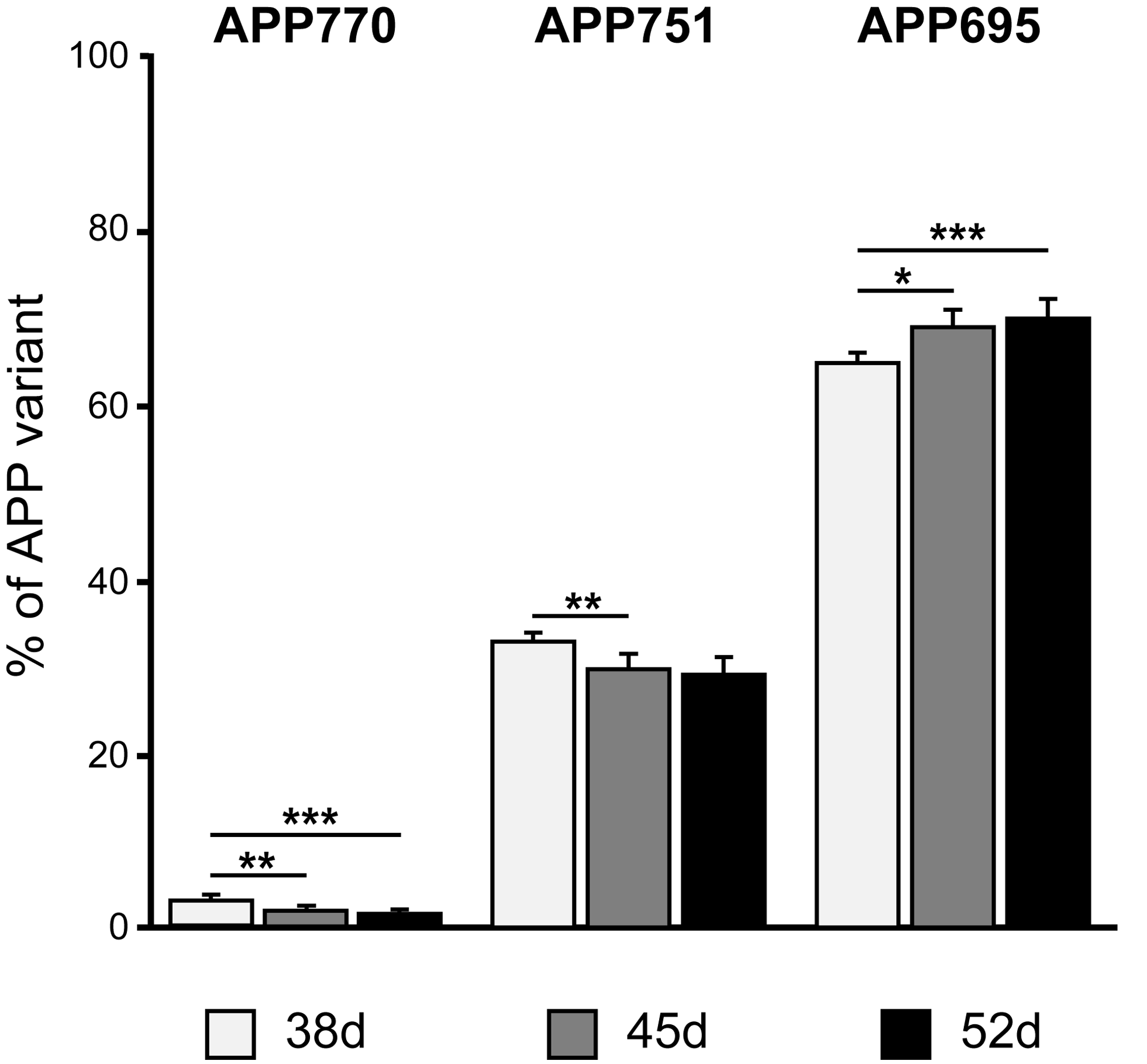

Supplement: Figure S2 — Percentages of the three isoforms of APP (APP770, APP751, and APP695) at 38, 45, and 52 days. Each column represents mean ± SD of 8 assays. * p<0.05, ** p<0.01, *** p<0.001, Tukey's test. (TIF) [file pone.0025788.s002.tif]

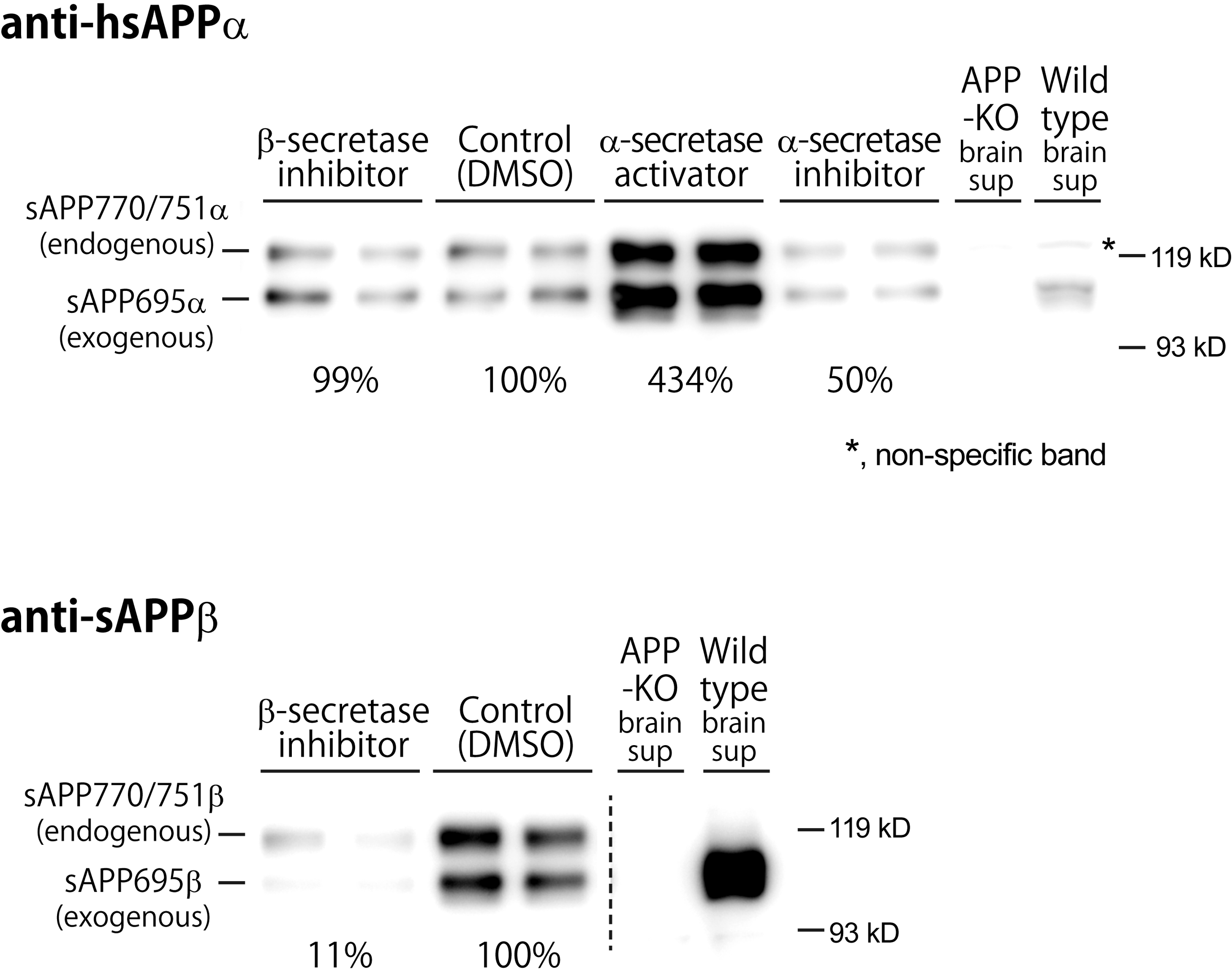

Supplement: Figure S3 — New hsAPPα and sAPPβ antibodies specifically detect human sAPPα and sAPPβ by western blots, respectively. Human neuroglioma H4 cells overexpressing wild-type APP (APPWT-H4 cells) were treated with α-secretase activator (12-O-tetradecanoylphorbol 13-acetate (TPA)), α-secretase inhibitor (TNF-α protease inhibitor-2 (TAPI-2)), or β-secretase inhibitor (see Protocol S1). Brain lysates of APP-knockout mice (APP-KO) were used as negative control. Immunoblots of conditioned media and supernatants of brain lysates were probed by anti-hsAPPα or anti-sAPPβ antibody. sAPPα or sAPPβ derived from both exogenous APP695 and endogenous APP770/751 are detected by each antibody. The increase in sAPPα by α-secretase activator and the reduction in sAPPα by α-secretase inhibitor effectively reached 434% and 50% of control (DMSO), respectively (upper panel). The decrease in sAPPβ by β-secretase inhibitor effectively reached 11% of control (lower panel). Neither sAPPα nor sAPPβ in the APP-KO brain was detected by anti-hsAPPα or anti-sAPPβ antibody, respectively. An asterisk indicates a non-specific band. (TIF) [file pone.0025788.s003.tif]

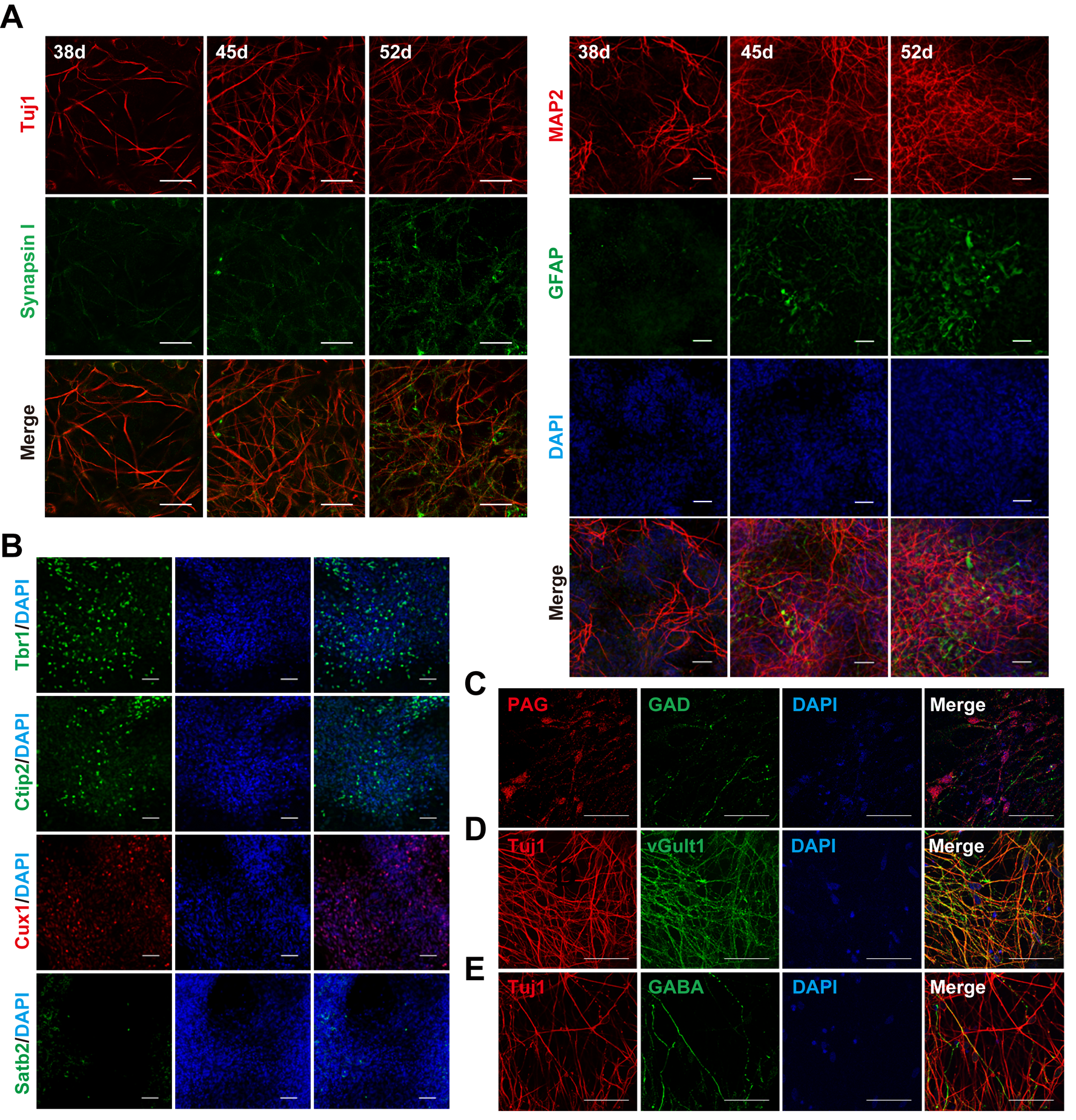

Supplement: Figure S4 — Immunocytochemical characterization of human ES cell (H9)-derived neuronal cells. (A) Time-dependent morphological changes of cells reseeded in a 24-well plate. Neuronal and glial cells were stained by anti-Tuj1 (left; red), anti-synapsin I (left; green), anti-MAP2 (right; red), and anti-GFAP (right; green) antibodies and DAPI (right; blue) at 38, 45, and 52 days. Scale bar, left; 20 µm, right; 50 µm. (B) ICC staining of Tbr1-, Ctip2-, Cux1- and Satb2-positive cells at day 52. (C–E) Neurotransmitter phenotypes at day 52. PAG (red)- and GAD (green)-positive (C), Glut1 (green)- and Tuj1 (red)-positive (D), and GABA (green)- and Tuj1 (red)-positive cells (E). Blue, DAPI. Scale bar, 50 µm. (TIF) [file pone.0025788.s004.tif]

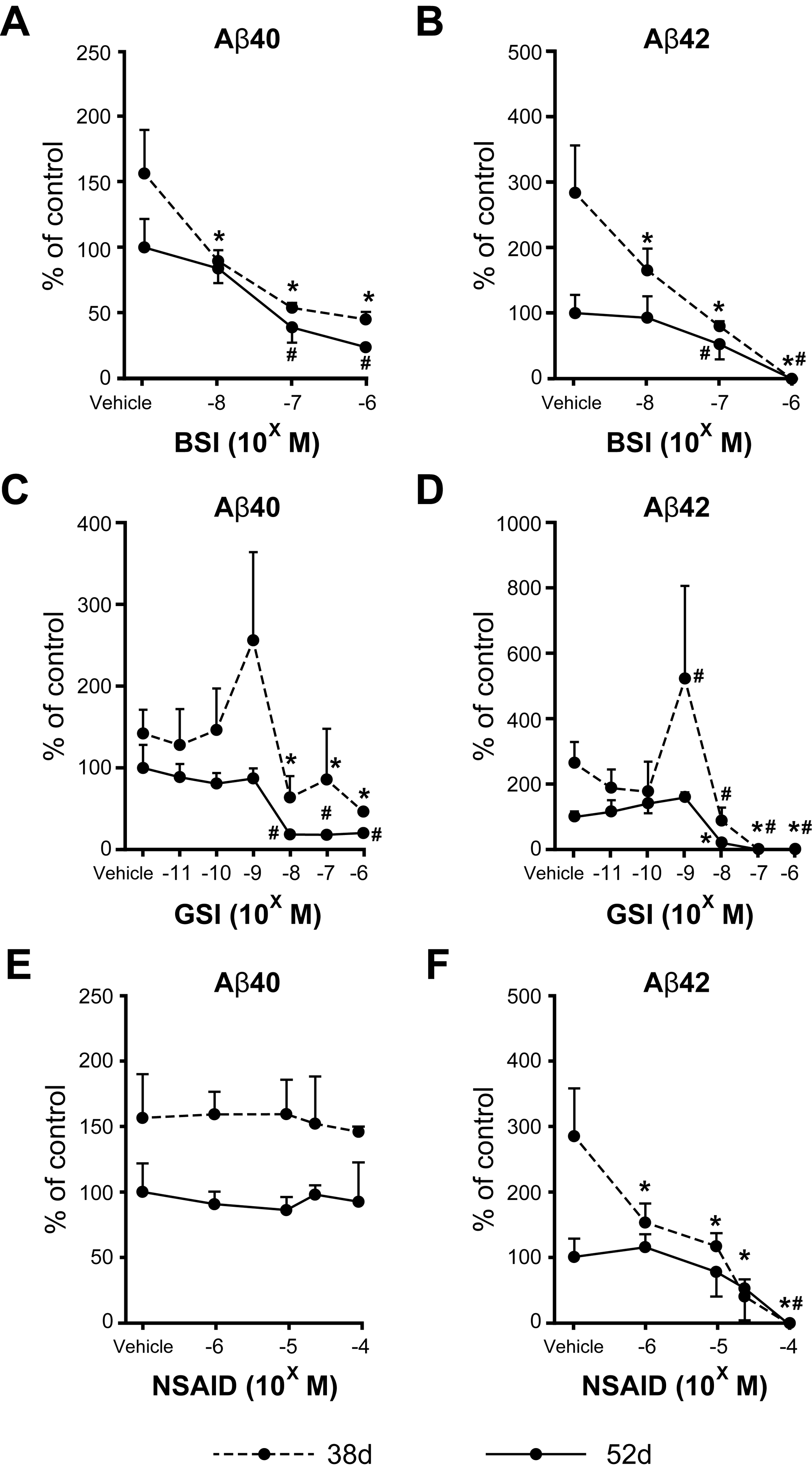

Supplement: Figure S5 — Aβ production was modulated by several drugs in human ES cell-derived neuronal cells. β-Secretase inhibitor (BSI) (A, B), γ-secretase inhibitor (GSI) (C, D), and NSAID (E, F) were added into hES cell-derived neuronal cell cultures at day 36 (dotted line) and 50 (bold line), and two days later amounts of Aβ40 and Aβ42 secreted into the conditioned media were measured. The ratios Aβ40/FL-APP and Aβ42/FL-APP are expressed as percentages of the vehicle-treated group at day 52 and represent mean ± SD of 3 assays. *, # p<0.05,**, ## p<0.01, ***, ### p<0.001, significantly different from respective vehicle-treated groups by Dunnett's test. (TIF) [file pone.0025788.s005.tif]

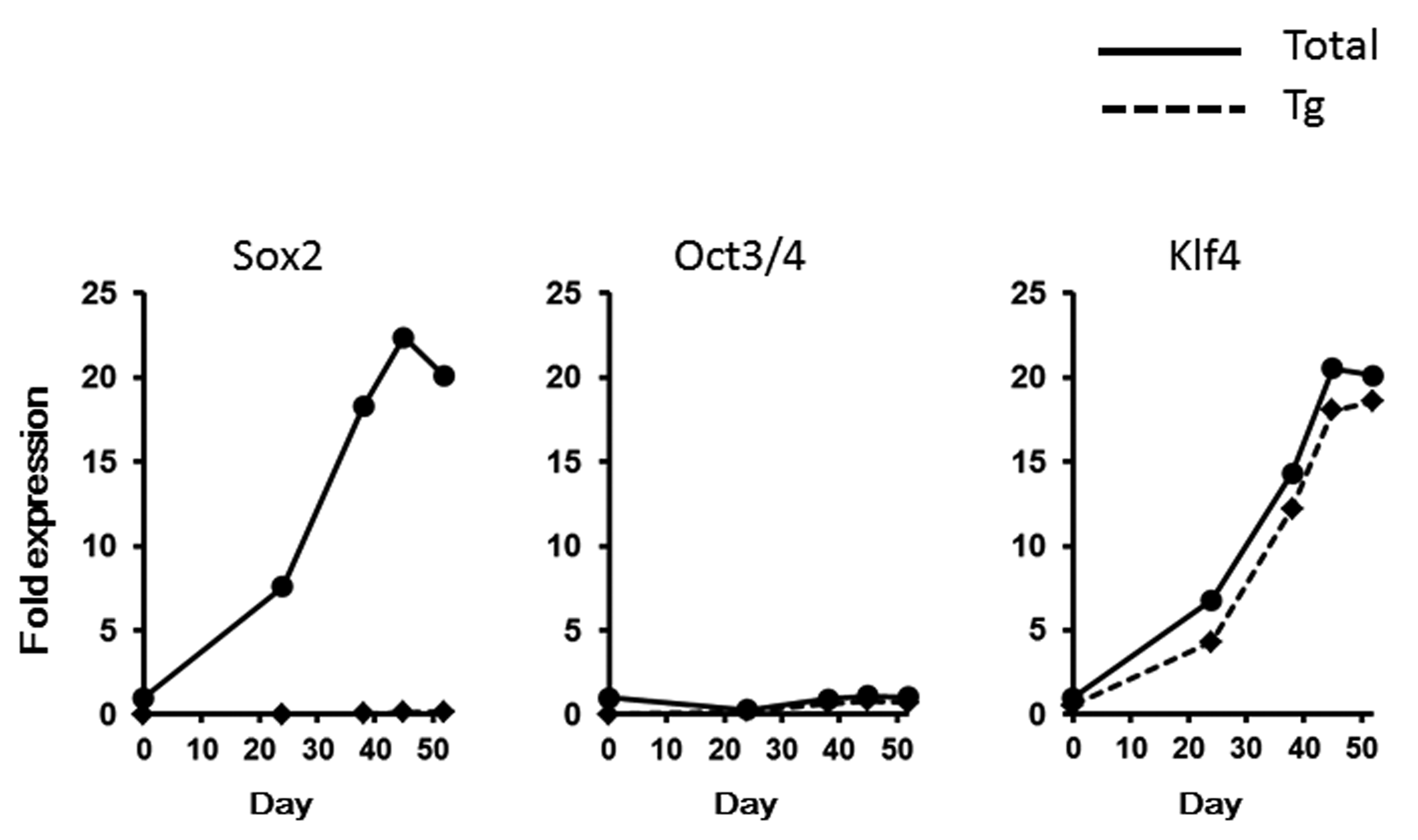

Supplement: Figure S6 — Expression levels of reprogramming factors of iPS cells in neural differentiation. Total and transgene (Tg) expression levels of Sox2, Oct3/4 and Klf4 were measured by qPCR. Bold and dotted lines represent total and transgene expressions, respectively. “Fold expression” represents the ratio of the expression level compared to the total expression level at day 0 (iPS cells). (TIF) [file pone.0025788.s006.tif]

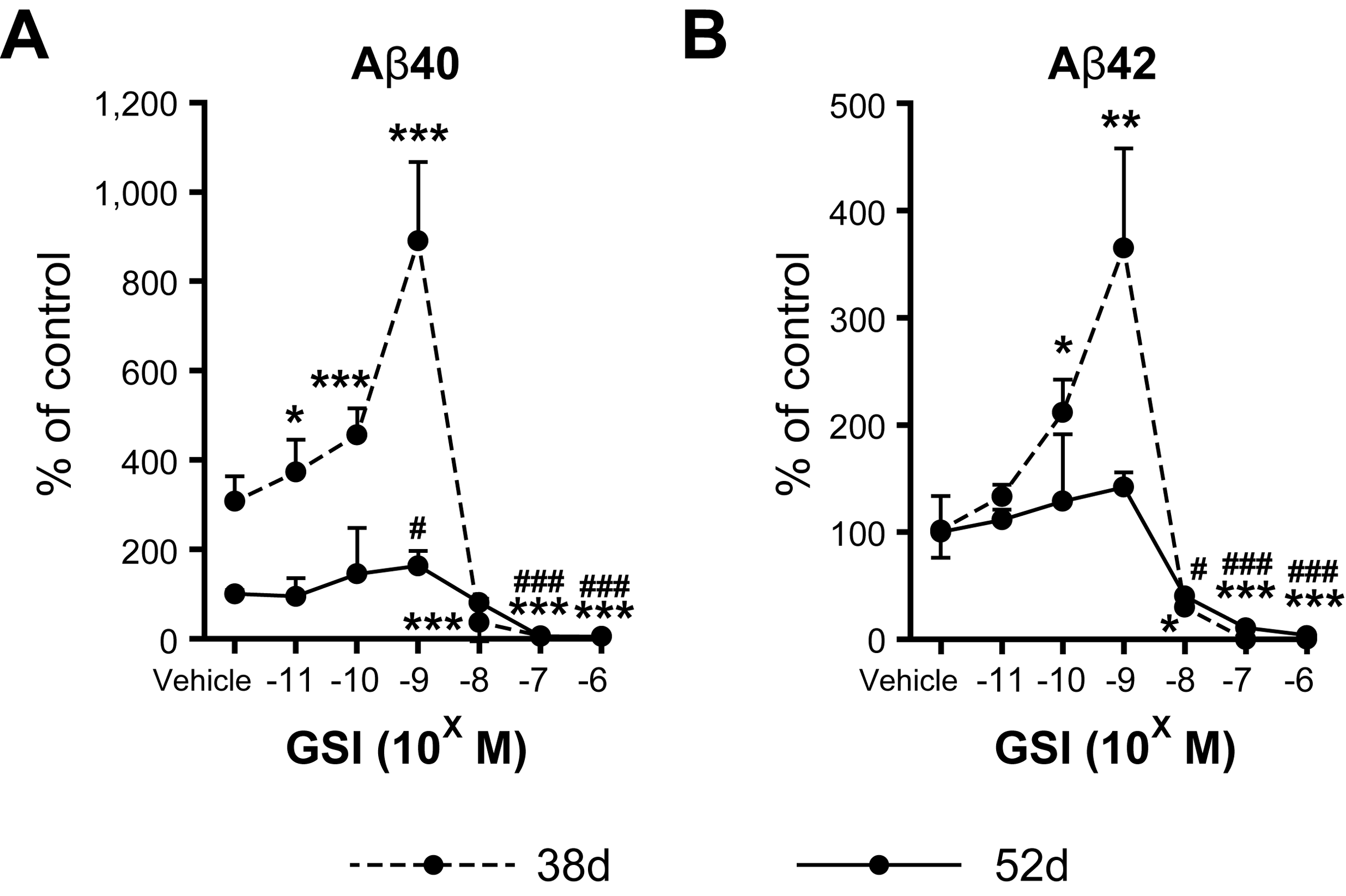

Supplement: Figure S7 — Aβ production was modulated by GSI in human iPS cell (201B7)-derived neuronal cells. γ-Secretase inhibitor (GSI) was added into the hiPS cell line, 201B7-derived neuronal cell cultures at day 36 (dotted line) and 50 (bold line), and two days later amounts of Aβ40 (A) and Aβ42 (B) secreted into the conditioned media were measured. The ratios Aβ40/FL-APP and Aβ42/FL-APP are expressed as percentages of the vehicle-treated group at day 52 and represent mean ± SD of 3 assays. (TIF) [file pone.0025788.s007.tif]
