# Supplementary material for: Anti-Aβ Drug Screening Platform Using Human iPS Cell-Derived Neurons for the Treatment of Alzheimer's Disease
Source: PLoS One. 2011 Sep 30;6(9):e25788. doi: 10.1371/journal.pone.0025788 (PMC3184175; doi:10.1371/journal.pone.0025788)
Supplement: Protocol S1 — Sampling method for checking antibody specificity. (PDF) [file pone.0025788.s008.pdf]

Anti-amyloid β drug screening for Alzheimer's disease treatment using human induced pluripotent stem cell-derived neuronal cells requires period of differentiation.

Naoki Yahata[1,2], Masashi Asai[2,3,4], Shiho Kitaoka[1,2], Kazutoshi Takahashi[1], Isao Asaka[1,2], Hiroyuki Hioki[2,5], Takeshi Kaneko[5], Kei Maruyama[3], Takaomi C. Saido[4], Tatsutoshi Nakahata[1], Takashi Asada[6], Shinya Yamanaka[1,7], Nobuhisa Iwata[2,4,8]* and Haruhisa Inoue[1,2,7]*

Supporting Information

Protocol S1

Sampling method for checking antibody specificity

Reagents

β-Secretase inhibitor IV, 12-*O*-tetradecanoylphorbol 13-acetate (TPA), and TNF-α protease inhibitor-2 (TAPI-2) were purchased from Merck (Darmstadt, Germany), Wako Pure Chemicals (Osaka, Japan), and Peptide Institute (Osaka, Japan), respectively. All reagents were dissolved in sterilized dimethyl sulfoxide (DMSO) and added to the cell culture medium to yield 0.1% DMSO as final concentration.

Cell culture

H4 neuroglioma cells stably expressing wild-type human APP_{695} (APP_{WT}-H4 cells) [1] (expression vector pCEP4; Invitrogen, San Diego, CA) were cultured in Dulbecco's modified Eagle's medium (Invitrogen, San Diego, CA) at 37°C with 5% CO_2. The medium was supplemented with 10% fetal bovine serum (Invitrogen), 100 U/mL penicillin, 100 μg/mL streptomycin (Invitrogen), and 150 μg/mL hygromycin B (Wako). APP_{WT}-H4 cells were plated at a density of 1.5×10^5 cells per 60-mm dish. After 48-h incubation, the conditioned media were replaced by new media containing the indicated reagent. Twenty-four hours after replacement of the media, the cultured media were harvested and precipitated with heparin agarose resin (Thermo Fisher Scientific, Rockford, IL), as described previously [2].

References

1. Asai M, Yagishita S, Iwata N, Saido TC, Ishiura S, et al. (2011) An alternative metabolic pwathway of amyloid precursor protein C-terminal fragments via cathepsin B in a human neuroglioma model. FASEB J, ***in press***

2. Asai M, Iwata N, Tomita T, Iwatsubo T, Ishiura S, et al. (2010) Efficient four-drug cocktail therapy targeting amyloid-β peptide for Alzheimer's disease. J Neurosci Res 88: 3588-3597.
