# Supplementary material for: Anti-Aβ Drug Screening Platform Using Human iPS Cell-Derived Neurons for the Treatment of Alzheimer's Disease
Source: PLoS One. 2011 Sep 30;6(9):e25788. doi: 10.1371/journal.pone.0025788 (PMC3184175; doi:10.1371/journal.pone.0025788)
Supplement: Table S1 — Effects of secretion inhibitors on cell viability measured by LDH assay at day 52. (DOCX) [file pone.0025788.s009.docx]

Table S1

Effects of secretion inhibitors on cell viability measured by LDH assay at day 52

| **Reagent [concentration]** | **LDH release (% of control)** | |
| --- | --- | --- |
|  | mean | ± SD |
| Positive control | 100.0 | ± 10.9 |
| Negative control (Vehicle) | 0.0 | ± 0.3 |
| β-Secretase inhibitor IV (BSI) [10^-6^ M] | -0.2 | ± 1.5 |
| Compound E (GSI) [10^-6^ M] | 0.1 | ± 1.7 |
| Sulindac sulfide (NSAID) [10^-4^ M] | 2.4 | ± 2.8 |

Each column represents mean ± SD of 3 assays.
