# Supplementary material for: Anti-Aβ Drug Screening Platform Using Human iPS Cell-Derived Neurons for the Treatment of Alzheimer's Disease
Source: PLoS One. 2011 Sep 30;6(9):e25788. doi: 10.1371/journal.pone.0025788 (PMC3184175; doi:10.1371/journal.pone.0025788)
Supplement: Table S2 — qPCR primers. (DOCX) [file pone.0025788.s010.docx]

Table S2

qPCR primers

| **Gene** | **Forward** | **Reverse** |
| --- | --- | --- |
| Foxg1 | tactaccgcgagaacaagca | tcacgaagcacttgttgagg |
| Cux1 | cagatgtccaccacctcaaa | ggtcaaataattctgttcgagtttt |
| Satb2 | cctcctccgactgaagacag | tggtctgggtacaggcctac |
| Tbr1 | gcacaagcagcaagatcaaa | caaccagcaaatgcttctca |
| Ctip2 | atcctcagccccttttgttt | gccgttgttcctgaattgtt |
| Tuj1 | aacgaggcctcttctcacaa | ggcctgaagagatgtccaaa |
| GFAP | ggttgagagggacaatctgg | aggttgttctcggcttcca |
| Synapsin I | gacggaagggatcacatcat | ctggtggtcaccaatgagc |
| MAP2 | caggtggcggacgtgtgaaaattgagagtg | cacgctggatctgcctggggactgtg |
| BACE1 | gggcagggctactacgtg | cagcacccactgcaaagtt |
| Aph-1A | gcttcgcgttatcatcctg | ccaagatgaaccagaccaca |
| Aph-1B | aagtggggcatcctccttat | tgacgccaggtttattccata |
| ChAT | agctgtggaagaaagcctca | tgggaggtgaaacctagtgg |
| VAChT | tacggagagcgaagacgtgaa | atcatggctatgccagacgtg |
| Sox2 transgene | ttcacatgtcccagcactaccaga | gacatggcctgcccggttattatt |
| Sox2 total | ttcacatgtcccagcactaccaga | tcacatgtgtgagaggggcagtgtgc |
| Oct3/4 transgene | gctctcccatgcattcaaactga | cttacgcgaaatacgggcagaca |
| Oct3/4 total | ccccagggccccattttggtacc | acctcagtttgaatgcatgggagagc |
| Klf4 transgene | ccacctcgccttacacatgaaga | gacatggcctgcccggttattatt |
| Klf4 total | catgccagaggagcccaagccaaagagggg | cgcaggtgtgccttgagatgggaactcttt |
